# Neural correlates of early adversity among Bangladeshi infants

**DOI:** 10.1038/s41598-019-39242-x

**Published:** 2019-03-05

**Authors:** Sarah K. G. Jensen, Swapna Kumar, Wanze Xie, Fahmida Tofail, Rashidul Haque, William A. Petri, Charles A. Nelson

**Affiliations:** 1 Boston Children’s Hospital, Boston, Massachusetts, USA; 2 Harvard Medical School, Boston, Massachusetts, USA; 3 ICDDR, B, Dhaka, Bangladesh; 4 University of Virginia, Infectious Diseases & International Health, Charlottesville, Virginia USA; 5 Harvard Graduate School of Education, Cambridge, Massachusetts, USA

## Abstract

In this paper we explore the relationship between the Visual Evoked Potential (VEP), a component of the electroencephalogram elicited by visual stimuli, and cognitive functions in children growing up in an urban slum in Bangladesh. VEPs in response to pattern-reversing checkerboards were collected in 6 month-old-infants (n = 91) and 36-month-old children (n = 112). We examine variation in the amplitude and latency of the first positive component, the P1, of the VEP in relation to cognitive scores on the Mullen Scales of Early Learning and the Wechsler Preschool and Primary Scale of Intelligence. We also examine whether children’s caregiving experiences prior to the neuro-cognitive assessment explain variation in the P1 of the VEP. We find that the P1 amplitude of the VEP is related to concurrent cognitive performance in each respective cohort. We also find that the P1 amplitude at 6 months is prospectively associated with cognitive outcomes at 27 months, and the P1 amplitude at 36 months is prospectively associated with children’s IQ at 60 months. We find no associations between caregiving experiences and variation in the P1 of the VEP at 6 months, yet caregiving experience do explain variation in the P1 amplitude at 36 months. Caregiving experiences also explain variation in children’s concurrent and prospective cognitive functioning. The VEP may be used as a biomarker to index the neurobiological embedding of early adversity, which in turn may impact children’s cognitive functions.

## Introduction

Growing up in a resource-poor home is a known risk factor for compromised developmental outcomes across a range of domains including executive functions, language, and memory^1–3^. Numerous studies from both high and low-income countries have found that part of the association between poverty and poor child outcomes is driven by characteristics of the home environment including the amount of cognitively stimulating activities and language a child is exposed to^4,5^. Moreover, characteristics of the home environment have been shown to mediate some of the effects of poverty on neural outcomes that may underlie disparities in cognition, such as lower hippocampal volumes and dispersed electrophysiological activity during selective attention^6,7^. Finally, a psychosocial intervention study found that a parent-focused intervention targeting family stress regulation, responsive parenting, language use, and facilitation of child attentions was associated with altered neural responses and improved selective attention highlighting the importance of children’s social family environment for both cognitive and neurodevelopmental outcomes^8^. The current study utilizes the visual evoked potential (VEP) to interrogate brain functioning in a sample of children growing up in impoverished homes in an urban slum in Dhaka, Bangladesh.

The VEP is an exogenous component of ongoing EEG, generated in the visual cortex in response to visual stimulation^9^. The VEP is recorded non-invasively using scalp electrodes and the ease of assessment has made the VEP widely used in both clinical practice and research^9^. The VEP response can be observed early in life and becomes relatively mature by the first year^10^. The most commonly used quantification of the VEP is extraction of the amplitudes and latencies of the first three peaks, namely the first negative peak (N1), the first positive peak (P1), and the second negative peak (N2). The two later peaks, especially the P1, are generally believed to reflect higher-order visual processes compared with the early N1, which reflects the activity of afferent fibers when the sensory signal travels from the eye to the visual cortex^10^. Interindividual variation in the amplitude and latency of the VEP peaks has been examined mostly in relation to visual processing^11^, but may also reflect more global neural “efficiency” or “maturation”^12^. The VEP, recorded shortly after birth has, for instance, been used to make predictions about neurological and cognitive outcomes after pre- and perinatal complications including intrauterine growth restriction, maternal drug misuse during pregnancy, prematurity, and birth asphyxia^11,13,14^. Studies of children, adolescents, and adults have also observed relationships between VEP latencies and amplitudes and IQ, such that a higher IQ is related to faster latencies and larger amplitudes in the P1 and N2 components^12,14–18^.

**Figure 1:**
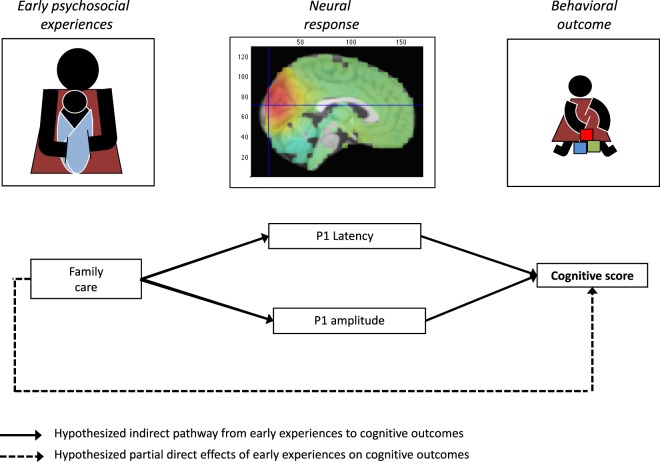
Hypothesized model.

Despite previous evidence linking variation in the VEP to concurrent and long-term cognitive outcomes, no previous study has, to the best of our knowledge, examined how psychosocial experiences such as characteristics of the caregiving environment may influence the VEP. Previous studies have noted that perinatal risks that are associated with altered VEP responses often co-occur with psychosocial risks such as poverty, and that psychosocial factors related to poverty therefore may contribute to observed variation in the VEP^11,13,14^. Emphasizing the importance of the early environment for the maturation of the VEP response, a recent study in rodents found that early impoverishment (reduced sensory and motor stimulation) led to alterations in the VEP^19^, yet no study has followed up on this association in humans^13,20^. Building on the previously shown associations between the VEP and cognitive outcomes in children exposed to poverty and related risks, and the evidence for associations between characteristics of early stimulation (enrichment) and the VEP in rodents, the current study examines the hypothesis that psychosocial exposures related to children’s caregiving experiences predict variation in the VEP, which in turn predicts variation in children’s early cognitive outcomes (Fig. 1). We also test the hypothesis that the association between the VEP and children’s behavioral outcome will be strongest for “visual reception” – a subscale of the Mullen Scales of Early Learning (MSEL) that is related to visual and higher order cognitive functions as opposed to the VEP being more broadly associated with early motor and language outcomes. We use data from two cohorts of children living a poor urban neighborhood in Dhaka, Bangladesh. The youngest cohort was 6 months old when VEPs were assessed, and cognitive outcomes were assessed using the MSEL at 6 and 27 months. The older cohort was 36 months old when VEPs were assessed, and cognitive outcomes were assessed using the MSEL at 36 months and the Wechsler Preschool and Primary Scale of Intelligence (WPPSI) at 60 months. Family caregiving activities were assessed at the time of the neurocognitive assessments; mothers reported caregiving activities in the 30 days prior to the neurocognitive assessment in both cohorts. We focus on the P1 component of the VEP, which was recorded from an occipital electrode over the visual cortex in response to transient pattern-reversing checkerboards. Previous research suggests that the P1 is the most consistent feature of children’s VEP response that is present from birth^21^, but stabilizes over the first few years^10^. Moreover, the P1 may be the VEP component most closely related to cognitive functions^10^. The current study explores whether the VEP can be used as a more objective and possibly culture- and language-free measure of a child’s neurocognitive development to complement behavioral measures of cognition, which may be impacted by societal norms and language capabilities. Compared with commonly used behavioral measures, the VEP may be particularly useful with infants and young children who have a limited behavioral repertoire.

## Methods

### Study design

Data were collected at our neuroimaging laboratory in Dhaka, Bangladesh. Children from the “Cryptosporidium Burden Study” (Crypto) cohort (N = 130) were 6 months old at the time of the VEP and first cognitive assessment and underwent a second cognitive assessment around the age of 27 months. Children from the “Performance of Rotavirus and Oral Polio Vaccines in Developing Countries” (PROVIDE) cohort (N = 130) were 36 months old at the time of the VEP and first cognitive assessment and underwent a second cognitive assessment at 60 months. Exclusion criteria limited the sample to infants and children born > = 34 weeks gestation, with no history of neurological abnormalities or traumatic brain injury, no known genetic disorders, and no known visual or auditory delays or impairments. Descriptive statistics are shown in Table 1. Ethical approval for the study was obtained from research review and ethics review committees at The International Centre for Diarrheal Disease Research, Bangladesh and Institutional Review Boards at Boston Children’s Hospital and were in accordance with local guidelines and regulations. All families provided informed, written consent to participate in the study.

**Table 1:** Sample descriptive information.

	6 months	27 months	36 months
**Child characteristics**			
Mean age at cognitive assessments	6.16 [0.14]	27.01 [2.30]	36.15 [0.49]
Mean gestational age	37.92 [1.83]		37.30 [1.31]
Preterm/ Born<37 weeks gestation	25%		41%
**Cognitive scores**			
MSEL Early learning (cognitive) composite score	34.79 [2.25]	94.16 [9.58]	119.68 [7.87]
Gross motor raw score	10.02 [1.19]	25.21 [2.11]	28.34 [1.56]
Visual reception raw score	11.10 [1.22]	24.55 [3.25]	31.65 [3.02]
Fine motor raw score	9.11 [0.84]	23.78 [2.77]	30.40 [1.07]
Receptive language raw score	8.54 [1.02]	24.32 [2.60]	29.31 [2.25]
Expressive language raw score	6.05 [0.38]	21.27 [3.151]	28.32 [3.38]
**Socioeconomic characteristics**			
Household income pr. household member pr. day	105.16 [48.38]		87.31 [68.55]
Average number of housing risks	3.43 [1.45]		3.79 [1.550
Average number of assets	6.06 [1.67]		5.65 [1.98]
**Family caregiving activities and frequencies in which caregivers engaged in each activity in the last 30 days [% yes]**			
Family care score	4.70 [2.10]		8.96 [3.04]
Play activities with toys that make music	17%		63%
Play activities involving drawing or writing	10%		89%
Play activities pretending to be someone else	14%		92%
Play activities encouraging movement	13%		98%
Play activities teaching shapes and colors	24%		25%
Reading activities	25%		56%
Telling stories or nursery rhymes	62%		71%
Singing	71%		54%
Play activities with toys	83%		63%
Counting or drawing	6%		50%
Playing using fingers, arms and/or legs	66%		57%
Chatting with child	41%		76%
Does child have access to books with pictures?	17%		67%
Does child have access to magazines with pictures?	23%		34%

Shown as “Mean [SD]” or percentage.

### Measures

#### Recording and processing of visual evoked potentials (VEPs)

Visual evoked potentials (VEPs) were acquired as part of a larger test battery that included resting state EEG and event-related potentials. During data collection children were seated on their caregiver’s lap in a dimly lit room in front of a monitor with an attached Tobii X2-60 eye-tracking system at a viewing distance of 65 cm from the screen. Pattern-reversal VEPs were recorded via an Electrical Geodesics, Inc. (EGI, Eugene, OR) system using a 128-channel high density HydroCel Geodesic Sensor Net (EGI, Eugene, OR) and amplified with a NetAmps 300 high-input amplifier. Data were sampled at 500 Hz. Presentation of the stimuli on the monitor was managed by E-Prime software (Psychology Software Tools, Pittsburgh, PA). Stimuli were presented for 500 ms and the stimulus phase-reversal was driven by the infants’ visual fixation on the screen and monitored by the Tobii Eye-Tracking system such that stimuli reversal continued only as long as the infants’ gaze was fixated on the screen (minimum fixation time 100 milliseconds).

EEG signal processing was done offline in NetStation 4.5. The EEG data were filtered with a 0.3–30 Hz finite impulse response (FIR) band-pass filter and segmented from 100 ms before stimulus onset as the baseline to 300 ms following stimulus onset, and the segments were then baseline corrected. Automated artifact detection was applied to detect channels within each segment that had a voltage change exceeding 200 μV. The VEP data were extracted from the occipital midline electrode 75 (Oz), referenced to the vertex electrode (Cz) at acquisition, and re-referenced using the average reference. Data were averaged across all trials for each subject. As per the International Society for Clinical Electrophysiology of Vision standard^9^, the amplitude of the P1 was measured relative to the preceding N1 peak (P1 – N1 amplitude/latency), within a time window from 70–120 ms. This time window was selected after visually inspecting the waveform from each individual subject. The window was selected to be inclusive of the P1 for all subjects. A trial was rejected if electrode 75 (Oz) was marked bad, or if it contained eye blinks, eye movements, or high-frequency noise as determined by visual inspection of each segment. Because the number of trials included in the average may affect the degree of noise in the data^22^, we used an inclusion criterion of a minimum of 20 trials per subject,which was met for all subjects. The 6 months old infants viewed between 32 and 100 trials (mean = 90.63, SD = 17.78), and the 36-month-olds viewed between 40 and 100 trials (mean = 94.80, SD = 13.95). After preprocessing of the VEP data we had useable data from n = 91 6-month-olds and n = 112 36-month-olds.

#### Cognitive assessments

Children’s cognitive development was assessed using an adapted version of the MSEL^23^ at 6, 27, and 36 months, and using the WPPSI at 60 months. The MSEL is an interactive assessment of child development and provides a global measure of cognition (the early learning composite score) and 5 subscale scores: gross motor, fine motor, visual reception, receptive language, and expressive language. The MSEL was developed in the US, but has previously been used in low-income countries^24,25^. Local staff adapted the protocol by substituting unfamiliar images and questions with objects and examples that Bangladeshi children would recognize. Given the absence of local norms for children in Bangladesh we standardized children’s raw scores within the sample in each cohort and for each time point to obtain culturally and age appropriate standardized z-scores. The composite scores were computed as the z-score of the summed subscale raw scores.

Similar to the MSEL, the WPPSI is an interactive assessment that provides both a global and subscale assessments of children’s cognitive development. The WPPSI was developed and normed in the US, but has previously been used to assess cognitive outcomes in 5 year old children in Bangladesh^26,27^. It was administered at 60 months of age in the oldest cohort to avoid potential ceiling effects on the MSEL. Here we focus on the full-scale intelligence quotient (IQ) and two subscales, namely verbal IQ and performance IQ.

#### Family caregiving environment

Family caregiving activities were assessed through oral interviews with mothers at the time of the first neurocognitive assessment (VEP and cognition) using the Family Care Indicators (FCI). The interviews were conducted by local, native staff. The FCI has been widely used in low and middle-income countries including Bangladesh^28^. The FCI uses items from UNICEF’s Multiple Indictor Cluster Survey as well as additional questions to assess stimulating activities that the mother, father, or an “other caregiver” engaged in with the child within the last 30 days (see Table 1).

### Statistical analyses

The hypothesized multivariate model (Fig. 1) was tested using structural equation modeling in Mplus version 7.4^29^. Model A explored relationships between family care, variation in the P1 (amplitude and latency), and children’s global cognitive score (the MSEL composite score or full-scale IQ). To test whether associations between variation in the VEP and global cognition were driven by specific subscales, an exploratory Model B substituted the global cognitive score for specific domain scores. Model fit was evaluated based on common guidelines with acceptable fit indicated by a non-significant *X*^2^ (p > 0.05), the confirmatory fit index (CFI) >0.95, the root mean square residual (SRMR) < 0.08, and RMSEA < 0.06^30^.

Indirect effects through which family care may impact variation in the P1 (latency or amplitude), which in turn may impact children’s cognitive outcomes, were estimated using the MODEL INDIRECT command in Mplus. We bootstrapped 10,000 times with bias-corrected confidence intervals. Missing data on the independent variables was replaced using full information maximum likelihood estimation with robust standard errors. Only children with non-missing VEP and cognitive data were included in the analyses leaving n = 91 with 6 months MSEL outcomes, n = 74 with 27 months MSEL outcomes, n = 112 with 36 months MSEL outcomes, and n = 106 with WPPSI outcomes at 60 months.

## Results

### Description of the visual Evoked Potential (VEP) waveform

**Figure 2:**
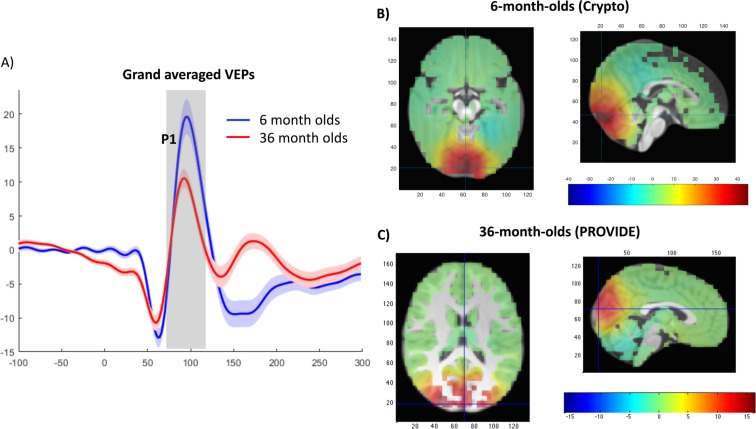
Grand averaged VEP. (**A**) Grand averaged VEPs recorded from the Oz electrode (electrode 75) for the 6- and 36-month-old cohorts; the shade areas represent the 95% confidence intervals of the amplitude across participants. (**B**) Cortical maps showing the neural source of the P1 in the 6-month-olds; (**C**) Cortical maps showing the neural source of the P1 in the 36-month-olds (see supplemental material for details).

Visual inspection confirmed the prototypical VEP waveforms, including the N1, P1, and N2 pattern for all participants. Grand averages for the 6- and 36-month-olds are shown in Fig. 2A. As expected, the N1 occurred between 40 and 100 ms, P1 occurred between 70 and 120 ms, and the N2 occurred between 100 and 170 ms after stimulus onset. To confirm the anatomical source of the neural activity underlying the P1 we conducted a source modeling analysis (see supplemental methods), which showed that the P1 was generated over the visual cortex in the occipital lobe in both cohorts (see Fig. 2B,C).

### Correlations

Bivariate correlations between study variables are shown in Table 2. In the 6 months old infants, there were no correlations between family care and neither the amplitude nor the latency of the P1 of the VEP. There were also no significant correlations between the P1 amplitude or latency and children’s concurrent cognitive composite or developmental domain scores in the 6 months old infants. The P1 amplitude at 6 months did, however, correlate with children’s cognitive composite score and all developmental domain scores, except for gross motor, at 27 months.

**Table 2:** Bivariate correlations between children's cognitive scores, the VEP components (the P1 amplitude and latency), family care, and gestational age.

	Peak P1 amplitude (μV)	Latency to peak P1 (ms)	Family care	Gestational age
**6-month-old cohort (Crypto)**				
**MSEL outcomes at 6 months (n** = **91)**				
Cognitive composite score	0.186°	0.109	−0.075	0.119
Gross motor raw score	0.118	0.129	0.039	0.221*
Visual reception raw score	0.148	0.097	−0.166	0.092
Fine motor raw score	0.050	−0.012	−0.045	0.044
Receptive language raw score	0.192°	0.097	0.147	0.103
Expressive language raw score	−0.009	0.095	−0.200	0.033
**MSEL outcomes at 27 months (n** = **74)**				
Cognitive composite score	**0**.287**	−0.037	0.113	**0**.255*
Gross motor raw score	0.172	−0.006	0.123	**0**.225*
Visual reception raw score	**0**.255*	0.040	0.114	**0**.233*
Fine motor raw score	**0**.206*	−0.143	0.048	**0**.289**
Receptive language raw score	**0**.306**	−0.018	0.031	0.155
Expressive language raw score	**0**.205*	−0.012	0.148	0.180°
**Psychosocial experiences (n** = **91)**				
Family care	−0.044	−0.068	—	—
Gestational age	0.105	0.121	0.052	—
**36-month-old cohort (PROVIDE)**				
**MSEL outcomes at 36 months (n** = **112)**				
Cognitive composite score	**0**.291**	−0.003	**0**.237*	−0.006
Gross motor raw score	0.061	−0.010	0.147	0.065
Visual reception raw score	**0**.252**	−0.026	**0**.258**	0.015
Fine motor raw score	**0**.383**	0.023	**0**.226*	0.103
Receptive language raw score	**0**.192*	−0.036	**0**.264**	−0.072
Expressive language raw score	0.084	0.032	0.008	−0.048
**WPPSI outcomes at 60 months (n** = **106)**				
Full scale IQ	**0**.249**	−0.002	**0**.277**	−0.090
Performance IQ	**0**.260**	0.013	**0**.204*	−0.036
Verbal IQ	**0**.207*	−0.064	**0**.276**	−0.113
**Psychosocial experiences (n** = **106)**				
Family care	**0**.208*	0.136	—	—
Gestational age	0.099	−0.045	0.039	—

μV = micro volt; ms = milliseconds. °p < 0.1; *p < 0.05; **p < 0.01. MSEL = Mullen Scales of Early Learning. WPPSI = Wechsler Preschool and Primary Scale of Intelligence

In the 36-months-old children, we found that family care was positively correlated with the P1 amplitude at 36 months. The P1 amplitude at 36 months was also positively correlated with children’s concurrent cognitive composite score, and with three developmental domain scores, namely visual reception, fine motor, and receptive language. The P1 amplitude at 36 months was also correlated with full scale IQ, verbal IQ, and performance IQ at 60 months. There were no correlatons between P1 latencies and either familie care or cognitive outcomes.

### Path analyses predicting children’s cognitive outcomes based on the VEP

#### The 6-month-old cohort (CRYPTO)

The first set of models examined the hypothesized associations between family care and variation in the VEP and MSEL scores at 6 months. Model 1A, which included children’s cognitive composite score at 6 months as the outcome, showed good model fit (χ^2^(3) = 1.143, P < 0.614; CFI = 1.000; SRMR = 0.026; RMSEA = 0.000), and there was a positive association between the P1 amplitude and children’s cognitive composite score at 6 months. There were no associations between family care and either variation in the P1 amplitude or latency, or between family care and the cognitive composite score. There was also no association between the P1 latency and the cognitive composite score.

The exploratory Model 1B, which included the five domain scores as outcomes instead of the cognitive composite score, showed good model fit (χ^2^(9) = 5.320, P = 0.806; CFI = 1.000; SRMR = 0.024; RMSEA = 0.000), yet none of the associations between variation in the P1 amplitude or latency and children’s developmental domain scores reached significance. Family care also showed no association with the either the P1 amplitude, P1 latency, or any of the developmental domain scores.

**Figure 3:**
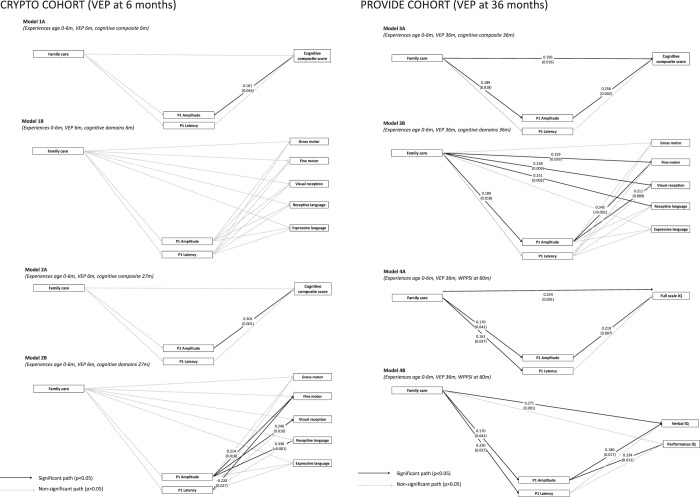
Multivariate model. The multivariate models showing all hypothesized path (gray and black), highlighting significant relationships in black, and providing standardized estimates and p-values for significant effects. Models “A” included the cognitive composite or full-scale IQ as the outcome while models “B” included the developmental domain/ subscale scores as the outcomes. All models corrected for potential effects of gestational age on the VEP and cognition, and the number of VEP trials was included as a covariate that may impact VEP amplitudes and latencies. m = months.

Model 2A (Fig. 3), which included the prospective cognitive composite score at 27 months as the outcome showed acceptable model fit, (χ^2^(3) = 1.112, P = 0.338; CFI = 1.000; SRMR = 0.044; RMSEA = 0.041). In this model, we found that higher P1 amplitudes at 6 months were related to higher cognitive composite scores at 27 months. Similar to models 1A and 1B there were no relationships between family care and variation in the P1 amplitude or latency, or between family care and cognition.

The exploratory Model 2B (Fig. 3), which used the five cognitive domain scores at 27 months as outcomes also showed acceptable model fit (χ^2^(7) = 8.251, P = 0.311; CFI = 0.992; SRMR = 0.051; RMSEA = 0.049). In this prospective model, we found that higher P1 amplitudes were related to higher fine motor, visual reception, and receptive language scores. Moreover, a shorter P1 latency was associated with better fine motor skills.

#### The 36-month-old cohort (PROVIDE)

Model 3A (Fig. 3) explored associations between family care, variation in the P1 (amplitude and latency) at 36 months, and children’s cognitive composite scores at 36 months. Model 3A showed acceptable model fit (χ^2^(3) = 4.942, P = 0.176; CFI = 0.945; SRMR = 0.044; RMSEA = 0.076). In this model, we observed a significant positive association between family care and the P1 amplitude. We also observed a positive association between the P1 amplitude and children’s cognitive composite score. Neither family care nor the cognitive composite score showed a significant relationship with the latency of the P1.

The exploratory Model 3B (Fig. 3), which examined the developmental domain scores as outcomes showed acceptable model fit (χ^2^(7) = 10.542, P < 0.160; CFI = 0.974; SRMR = 0.038; RMSEA = 0.067). In this model, we found that the P1 amplitude showed positive associations with fine motor and visual reception. We also saw direct effects of family care on visual reception and receptive language, independent of the effects of the VEP. There were no associations between the P1 latency and either family care or cognition.

Since family care predicted variation in the P1 amplitude at 36 months, and the P1 amplitude in turn predicted variation in the cognitive composite score, fine motor, and visual reception scores collected at 36 months, we explored the hypothesized indirect effect whereby caregiving experiences predict variation in the VEP, which in turn predict cognitive outcomes. Results are shown in Table 3. Only one indirect effect reached significance, namely the effect of family care on fine motor via the P1 amplitude of the VEP.

**Table 3:** Indirect effects of family care on cognitive outcomes via variation in the Visual Evoked Potential.

Risk exposure	Neural outcome	Behavioral outcome	Estimated effect	p-value	Bootstrapped 95% CI	
					Lower limit	Upper limit
**MSEL composite score at 36 months (n = 112)**						
Family care	P1 amplitude	Mullen composite	0.048	0.053	0.007	0.114
Family care	P1 amplitude	Visual reception	0.040	0.067	0.005	0.105
**Family care**	**P1 amplitude**	**Fine motor**	**0**.**065**	**0**.**045**	**0**.**009**	**0**.**143**
**WPPSI full scale IQ score at 60 months (n = 106)**						
Family care	P1 amplitude	Full scale IQ	0.037	0.102	0.001	0.100
Family care	P1 amplitude	Verbal IQ	0.031	0.132	0.000	0.091
Family care	P1 amplitude	Performance IQ	0.040	0.100	0.001	0.106

CI = Confidence Interval. MSEL = Mullen Scales of Early Learning. WPPSI = Wechsler Preschool and Primary Scale of Intelligence.

The prospective Model 4A with the VEP at 36 months predicting full-scale IQs at 60 months fit the data well (*χ*^2^(3) = 2.617, P < 0.455; CFI = 1.000; SRMR = 0.037; RMSEA = 0.000). Model 4A showed that the P1 amplitude at 36 months was positively related to full scale IQ at 60 months. Family care was positively associated with higher P1 amplitudes and greater P1 latencies, and there was a positive relationship between family care and full-scale IQ. There were no associations between P1 latencies and family care or full scale IQ. The indirect effect of family care on full scale IQ via variation in the P1 amplitude did not reach significance (see Table 3).

The exploratory Model 4B fit the data well (*χ*^2^(4) = 3.806, P < 0.433; CFI = 1.000; SRMR = 0.041; RMSEA = 0.000) and showed a positive relationship between the P1 amplitude and children's verbal and performance IQ. More family care was associated with higher P1 amplitudes, greater P1 latencies, and higher verbal IQ. None of the indirect effects of family care on children's verbal or performance IQ via the P1 reached significance (see Table 3).

## Discussion

The aim of this paper was to explore whether variation in the VEP can provide an early neural correlate of children’s cognitive abilities. In previous studies it has been found that VEPs recorded shortly after birth predict neurological and developmental outcomes in preterm babies^11,13^ and studies in children and adults have found correlations between variation in components of the VEP and concurrent IQ^15–17^. To the best of our knowledge, this is the first study to examine whether characteristics of children’s caregiving environment influences the VEP, which in turn may affect early cognitive outcomes.

Using data from two cohorts of infants and children from Bangladesh, we examined the hypothesis that VEPs measured at 6 and 36 months were associated with concurrent and later cognitive outcomes. We focused on the P1 component because this is the most consistent feature of infants’ VEP response that while it is present from birth^21^ continues to develop early in life^10^. Moreover, the P1 (latency and amplitude) has also been proposed to be the component of the VEP that is most closely related to cognitive functions^10^. We found significant positive associations between the amplitude of the P1 of the VEP and children’s concurrent and prospective cognitive composite score across four sets of models. Namely, we found that a higher P1 amplitude at 6 months was associated with higher cognitive composite scores at 6 and 27 months, and that a higher P1 amplitude at 36 months was associated with a higher cognitive composite score at 36 months and higher full-scale IQ at 60 months. In a set of exploratory analyses, we examined whether these associations were driven by specific subdomains of cognitive functions related to visual and attentional functions. Here we found that variation in the P1 amplitude at 6 months was unrelated to any specific developmental domain scores at 6 months, but prospectively associated with three developmental domain scores at 27 months, namely fine motor, visual reception, and receptive language. These findings suggest that the VEP may be related to broader developmental outcomes as opposed to being selectively related to attentional and visual functions reflected in the visual reception score. In the 36-months old children, we found that the P1 amplitude of the VEP was significantly related to concurrent visual reception and fine motor scores. The P1 amplitude at 36 months was also prospectively associated with higher verbal and performance IQ at 60 months. We saw one significant association between P1 latencies and child outcomes, namely a negative association between P1 latency at 6 months and fine motor scores at 27 months. This findings suggests that a faster P1 latency at 6 months was related to higher fine motor scores at 27 months. The finding of associations between variation in the P1 of the VEP - particularly the amplitude of the P1 - and children’s cognitive performance at all four time points supports our hypothesis that the VEP may provide a neural index of early cognitive functions.

With regard to potential mechanisms linking greater P1 amplitudes to better cognitive scores, we note that a recent study found that the P1 amplitude of the VEP is a good index of infants’ spatial and sustained attention, such that enhanced spatial and sustained attention is associated with an amplified P1 peak amplitude^31^. It is therefore possible that attention plays a role in the relationship between variation in the P1 and children’s cognitive outcomes. For example, infants with higher P1 amplitudes at 6 months might be better at allocating attention, which in turn facilitates the development of other high-level cognitive functions that can be detected later in life. At the neurobiological level, a larger versus a smaller P1 amplitude reflects more synchronous neural activity over the visual cortex in response to the stimulus (here, pattern reversal). A large P1 amplitude could therefore reflect more focal activity around the visual cortex which, in turn, may reflect greater synaptic efficiency in this region. Focal activation and increased synaptic efficiency could be indicative of functional specialization and developmental maturation of the visual area. Moreover, the VEP is likely influenced by myelination^21^. Myelination occurs rapidly over the first few years of life and serves to increase conduction velocity and again, synaptic efficiency. A recent study found that rodents exposed to low levels of stimulation showed delayed maturation of VEP latencies as indicated by higher VEP latencies at P28 and P31, roughly equivalent to a human age of 33–36 months. Moreover, changes in VEP latencies were accompanied by impaired motor and memory functions, as well as structural changes in the brain reflecting delayed myelination of nerve fibers in the visual cortex^19^. In humans, a combined DTI and VEP study found a strong correlation between the latency of the P1 and microstructural markers of myelination/ maturation^21^. Although the link between myelination and the latency of a neural response such as the VEP is most clear, it could be that myelination also impacts the amplitude of the neural response. We note that the association between higher P1 amplitudes and better cognitive scores should be considered relative to children’s age. Namely, as seen in Fig. 2, we observed that the amplitude of the P1 is substantially larger in the 6-month-olds relative to the 36-month-olds, suggesting that absolute size does not reflect neural maturation in an absolute manner across age-spans. The larger P1 observed in the 6-month-olds relative to 36-month-olds is likely due to increasing skull thickness with age or developmental changes in the location and direction of the sources of the P1 as seen in Fig. 2.

In the exploratory analyses we did not see any associations between variation in the P1 of the VEP and concurrent developmental subdomain scores in the 6 months old cohort, but we did see associations between variation in the VEP at 6 months and children's domain scores for visual reception, fine motor, and receptive language at 27 months. We also observed associations between concurrent P1 amplitudes and visual reception and fine motor domain scores in the 36-months-old children, and between P1 amplitudes at 36 months and verbal and performance IQ at 60 months. These exploratory analyses suggest that associations between the VEP and children’s cognitive outcomes were non-specific, but rather driven by or reflecting broader cognitive functions. Two methodological concerns may explain the lack of findings of associations between the P1 amplitude and domain scores in the 6-month-old infants. First, the absence of an association could reflect the smaller sample size in the 6 -month-old cohort compared with the 36-month-old cohort because fewer infants had usable EEG data used to estimate the VEPs. We note, however, that P1 amplitudes assessed at 6 months did show associations with three of the five subscales at 27 months, despite the smaller sample size, possibly suggesting that the lack of findings at 6 months is not caused solely by these analyses being underpowered. Another possible explanation for the lack of associations between variation in the VEP and MSEL scores at 6 months is that we, as expected, observed less variance in performance captured by the MSEL domain scores at 6 months compared with later in development where children are assessed across more items for each domain. The MSEL may therefore not provide enough differentiation between infants at 6 months. The VEP may also be a stronger predictor of prospective compared with concurrent cognitive functions because neural changes may emerge before, and possibly contribute to, subsequent variation in behavioral outcomes.

We did not observe any concurrent associations between family care and variation in the VEPs collected in infants at 6 months of age. We did, however, see associations between family care and P1 amplitudes in the 36-month-old children. The association between family care and the VEP in the 36-months-old children is consistent with previous studies in humans relating caregiving experiences to variation in brain structure and ERP responses during auditory attention^6,8^. A psychosocial intervention study mentioned above, for instance, found that a parent-focused intervention impacted children’s neural response during selective auditory attention such that children in the parent-focused intervention group showed more selective neural responses to attended versus unattended stimuli compared with children who received a child-focused or no intervention^8^. Moreover, children in the family-based intervention group also showed greater improvement of selective attention suggesting that changes in the ERP may contribute to better selective attention. The fact that we only see an association between the family care and the VEP at 36 months, and not 6 months, may suggest that environmental influences on neural functioning take time to emerge. This is in line with EEG studies showing associations between socioeconomic status and EEG power in 1 year old infant^32^, but not in neonates^33^.

Finally, in the 36-month-old children we explored indirect effects through which the amplitude of the P1 may impact cognitive outcomes via the effects of family care on the VEP. Only one indirect path, namely from family care to variation in the P1 amplitude at 36 months, and from the P1 amplitude to fine motor skills at 36 months reached significance. We note, however, that many of the indirect paths were approaching significance. It would therefore be interesting follow up on these indirect pathways in future research with larger samples that may enable better detection of indirect effects. Future studies may also add more age groups to further explore when the relationship between family care and P1 amplitudes begins to emerge.

The findings of the current study should be considered in light of a number of limitations. First, although the sample sizes used in the four sets of models is considerable for a neuroimaging study, the sample sizes are small for SEM models. Small samples sizes may cause complex models, such as the exploratory models, to be underpowered due to the number of estimated paths. As mentioned above, this is particularly true for the 6-month-old cohort where fewer infants had usable EEG data. Second, all results should be considered correlational since neither the concurrent associations nor the prospective associations are sufficient to address causality. Examination of causal relationships would require an experimental manipulation as seen in randomized controlled trials. Although we found evidence for one indirect effect from early caregiving experience to fine motor skills via the VEP we cannot be certain about the directionality of this effect. An alternative hypothesis could be that children’s developmental status (which may correlate with the P1) may impact parenting interactions because the types of stimulating activities parents engage in with a child may depend on the child’s cognitive (or motor) abilities. Third, we have limited information about perinatal events (gestational age) and although we excluded children born very preterm (< = 34 weeks gestation) or with known neurological abnormalities we cannot rule out that other perinatal complications that may correlate with both family care and the brain outcome may confound observed effects.

## Conclusion

A key aim of the current study was to explore whether the VEP can be used as an early neural marker of current and future cognitive outcomes in a community sample of infants and children living in an impoverished neighborhood in Dhaka, Bangladesh. We found consistent evidence for associations between P1 amplitudes and both concurrent and prospective cognitive functions in infants as young as 6 months. The association between the VEP and cognitive outcomes did not appear to be specific to the visual domain, but implicated visual/attention skills as well as motor and language functions.

We also explored the hypothesis that early caregiving experiences may predict variation in neural responses detected by the VEP, which in turn may be associated with children’s cognitive developmental outcomes. This hypothesis was confirmed for one indirect effect whereby more frequent caregiver-child interactions were associated with a magnified P1 response (higher P1 amplitude), which in turn was associated with better concurrent fine motor skills in the 36-month-old cohort.

Using the VEP as a complementary or alternative measure to cognitive functions in the assessment of children’s early development has several advantages over behavioral measures of cognition. First, the VEP may be considered a more objective assessment compared with behavioral assessments. The VEP may, for instance, be less impacted by cultural norms that can affect children’s understanding of complex instructions associated with many behavioral tasks. Moreover, a neural response like the VEP may be somewhat less impacted by subjective factors related to the person being tested (e.g., mood, arousal) and the tester (e.g., experience and biases). Second, the fact that VEP predicted prospective cognitive outcomes may suggest that the VEP can be used as a screening tool to enable earlier detection of whether a child is following an expected developmental trajectory. The prospective relationships may indicate that neural changes such as altered P1 responses may predispose variation in cognitive outcomes, and that characteristics of the VEP can be used to make predictions about a child’s prospective developmental outcomes. This, in turn, would suggest that the VEP may be used to identify children who stand to benefit most from interventions to support early cognitive development. One advantage of the VEP is that it is relatively simple to assess compared with other neuroimaging tools, and thus more easily incorporated into standard clinical or research practices, even in low resource settings like urban Bangladesh. More studies are needed to confirm associations between the VEP and cognitive outcomes, and to examine when associations between the VEP and caregiving experiences emerge.
